# Subtalar Arthroereisis with Calcaneus Stop Screws—Can the Angles on Pre- and Post-Surgical X-Ray Images Be Reliably Measured by Artificial Intelligence?

**DOI:** 10.3390/children12111552

**Published:** 2025-11-17

**Authors:** Lea Alexandra Simmler, Monika Herten, Samuel Hohenberger, Cedric Rubenthaler, Heinz-Lothar Meyer, Bastian Mester, Stephanie Herbstreit, Johannes Haubold, Manuel Burggraf, Marcel Dudda, Christina Polan

**Affiliations:** 1 Department of Trauma, Hand and Reconstructive Surgery, University Hospital Essen, 45147 Essen, Germany; lea.simmler@stud.uni-due.de (L.A.S.); samuel.hohenberger@ext.uk-essen.de (S.H.); cedric.rubenthaler@stud.uni-due.de (C.R.); heinz-lothar.meyer@uk-essen.de (H.-L.M.); bastian.mester@uk-essen.de (B.M.); stephanie.herbstreit@uk-essen.de (S.H.); marcel.dudda@uk-essen.de (M.D.); christina.polan@uk-essen.de (C.P.); 2 Institute for Artificial Intelligence in Medicine, University Hospital Essen, Girardetstraße 2, 45131 Essen, Germany; johannes.haubold@uk-essen.de; 3 Institute of Diagnostic and Interventional Radiology and Neuroradiology, University Hospital Essen, 45147 Essen, Germany; 4 Department of Orthopedics, Trauma, Hand- and Footsurgery, GFO Klinikum Mettmann-Süd, St. Martinus Hospital, 40764 Langenfeld, Germany; manuel.burggraf@gfo-kliniken-mettmann-sued.de; 5 Department of Orthopedics and Trauma Surgery, BG Klinikum Duisburg, 47249 Duisburg, Germany

**Keywords:** flat foot, calcaneus stop screw, foot X-ray image, AI measurement, foot angle measurement, subtalar arthroereisis

## Abstract

**Highlights:**

**What are the main findings?**

- AI was proven to deliver excellent results for angles in dorsoplantar plane, while in lateral view, AI either showed significant deviations from manual measurements or could only measure angles on a small proportion of X-rays.
- AI neither measured angles on preoperative X-rays significantly more often nor significantly better than on postoperative X-rays.
- AI measured angles on incorrectly taken radiographs at least as often as on correctly taken radiographs.

**What are the implications of the main findings?**

- AI still requires training in measuring angles on radiographs of children with severe flat feet on the lateral plane.
- AI was not disturbed by calcaneus stop screws.
- AI did not exclude incorrectly taken radiographs from angle measurements.

**Abstract:**

**Background/Objectives**: Flexible symptomatic flat foot in children can be surgically treated with calcaneus stop screws. This raises the question of whether pre- and postoperative radiographs (X-ray) can be analyzed in two planes using AI. **Methods**: In this monocentric retrospective study, angle measurements generated by Bone Metrics AI (Gleamer) were compared with manual measurements using Centricity™ (GE Healthcare). A total of 659 X-rays from 124 operated feet (2014–2024) were available, of which 422 were analyzable by AI and 299 met defined quality criteria. Bland–Altman plots were used to assess agreement. Linear and logistic regression analysis examined the influence of age, gender, accessory navicular bone, additional foot pathologies, and flat foot severity on comparability of the measurement methods and measurability by the AI. Finally, radiographs meeting and missing quality criteria were compared. **Results**: AI measurements were comparable to manual measurements for calcaneus inclination, hallux valgus, 1st–2nd and 1st–5th metatarsal angle both pre- and post-operatively. For the talus-1st metatarsal and medial arch angles, AI results differed significantly (*p* < 0.001 and *p* ≤ 0.013) from manual measurement. AI generated talus-1st metatarsal angle was measured larger by 6.14°, 95% [−7.14; −5.14] pre-operatively and 2.80°, 95% [−3.79; −1.81] post-operatively. Medial arch angle was smaller by 1.63° pre-operatively, 95% [1.03; 2.23] and 0.52° post-operatively, 95% CI [0.11; 0.93] with AI. Post-operative measurability was not significantly lower than pre-operative. AI measured angles on incorrectly taken radiographs as often or more often than on correctly taken ones. **Discussion:** Screw implantation did not negatively impair measurability or AI accuracy. However, age, gender, and flat foot severity influenced AI performance. Bad radiograph quality did not affect AI measurability negatively, indicating that AI cannot yet distinguish between X-rays suitable and unsuitable for angle measurements. **Conclusions**: Manual measurements are still indispensable in the diagnosis of children’s flat feet. In the future, continuous training of the AI is expected to bring it into line with manually measured radiological values.

## 1. Introduction

A flexible flat foot is defined as a flattening of the longitudinal arch combined with a valgus position of the hindfoot [1]. Unlike rigid flat foot, the arch reconstitutes during tiptoeing or passively extending the hallux (Jack-test) [2,3]. The flexible flat foot is highly prevalent in toddler age but typically improves naturally within the first decade of life due to muscular strengthening, ligament tightening, and tibial torsion [2,4,5,6,7]. However, in some children the arch fails to develop adequately. Risk factors include obesity, positive family history, neuromuscular disorders, joint hypermobility, genetic conditions, low physical activity, male sex, connective tissue disorders, lower extremity alignment disorders and wearing closed-toe shoes [2,5,6,7,8,9,10,11,12].

When lacking function or causing pain, pediatric flat feet become a condition requiring treatment [6]. Conservative management such as physiotherapy, targeted exercises, foot orthoses, insoles or orthopedic shoes often alleviate symptoms [2,6,12,13]. If conservative measures fail, surgical intervention is indicated to restore alignment and prevent long-term complications such as persistent pain, reduced endurance, or even a higher risk for metatarsal fractures [5,14,15,16]. The so-called calcaneus stop procedure is one of several surgical options indicated for symptomatic flat feet in children aged nine to thirteen [17]. It is a minimally invasive, cost-effective treatment method that directs growth and preserves function [14,15]. During the procedure, a screw is inserted into the bottom part of the sinus tarsi outside the canalis tarsi under radiological control, blocking excessive subtalar motion and preventing excessive calcaneal eversion and talar medial rotation [14,15]. The screw is left in place for a minimum of two to three years, or until the growth plates in the foot have closed, after which it is removed surgically [17]. Following its removal, the clinical or radiological improvements are preserved [17].

In addition to a detailed patient history and clinical assessments, radiographic measurements of foot angles in two X-ray planes before and after surgery are essential for both surgical planning and follow-up assessment [6,14,18]. Unfortunately, manual angle determination is time-consuming and subject to interobserver variability [19,20,21]. Artificial intelligence (AI)-based tools used in interpreting medical images have already proven enhanced and consistent precision in comparison to humans, thereby reducing misdiagnoses that could otherwise lead to unnecessary procedures [22]. By analyzing X-ray images more rapidly and by processing new information directly in combination with electronic health records, AI can be more precise and even more efficient than human diagnosticians. It can lead to earlier diagnoses resulting in earlier intervention [22].

Concerning foot angle measurements, AI-based tools have recently demonstrated high agreement with manual measurements and confirmed the time saving aspect of AI [21,23,24,25]. However, existing studies were limited by heterogeneous patient groups regarding age, prevalence of flat feet and surgical status, all together lacking focus on operated pediatric flatfoot [21,23,24,25]. To date, no study has evaluated AI-based angle measurement in pre- and postoperative radiographs of children treated with the calcaneus stop screw.

Therefore, the aim of the present study was to investigate whether AI-generated angle measurements were comparable to manual assessments in children with flexible flatfoot and if the implanted calcaneus stop screw had an influence on the measurement. Reasons that could have influenced the measurability or the comparability of the measurements were inspected. The ability of the AI to recognize X-rays that did not meet quality criteria was examined as well.

## 2. Materials and Methods

### 2.1. Data Collection

In this retrospective study, a total of 65 children with flexible flat feet who underwent surgery using calcaneus stop screws between January 2014 and August 2024, were included. The study was approved by the Ethic Committee of the Medical Faculty of Essen, Germany (24-11718-BO). Patient data were completely anonymized meaning that the patient’s parental consent was not required.

In six of these children, the procedure was performed unilaterally and in 59 children bilaterally, resulting in a total of 124 feet.

Radiological images were taken at three defined moments: pre-operatively (=pre), post-operatively during the inpatient stay (1st post-op, 1–14 days after surgery) (=inpatient), and post-operatively during the outpatient follow-up interval (2nd post-op, >14 days after surgery) (=post). A total of 659 X-ray images were taken in two planes, anteroposterior [AP] and lateral.

Patient characteristics were collected from clinical documentation in the Medico system and from radiological image analysis. The following details were included: the patient’s age at the time of the radiography, their gender, and the presence of either an accessory navicular bone (os tibiale externum (OTE) or os naviculare cornutum (ONC)) as well as the presence of other foot pathologies. These ranged from spread foot, pointed foot, sickle foot, pes cavovarus, hallux valgus, talus obliquus/verticalis, upper ancle joint valgus in ball talus, coalitio talonavicularis, os trigonum, talonavicular subluxation, serpentine foot, pes equinoplanus, accentuated posterior talar process, coalitio cuneiforme mediale–intermedium to os peroneum. Information on BMI, pain, range of motion, previous illnesses, previous operations, genetic disorders causing excessive ligamentous laxity, leg axis pathologies and pre-operative conservative therapy were extracted from the patient’s history. However, due to dependency on the examiner, incomplete documentation and the possibility of incomplete diagnostic of genetic comorbidities to this point, these data were not included in statistical analysis. As a result, we examined each X-ray in an unbiased manner without taking individual factors into account.

### 2.2. Manual Measurement

All images that met the quality criteria for a standardized X-ray image of the foot were measured manually by one medically well-trained person using Centricity Universal Viewer software 2024 (General Electric (GE) Healthcare GmbH, Düsseldorf, Germany). These measurements were defined as ground truth. The manually measured angles were supervised by a senior pediatric orthopedic surgeon and a senior radiologist.

### 2.3. AI Measurement

#### 2.3.1. AI Algorithm Screening

BoneMetrics (Gleamer, Saint Mandé, France, version 2.5.0.1) was the AI system used in this study. This CE-marked radiological fully automated image processing software worked through conventional radiographs across different anatomical regions, including the foot. BoneMetrics used a deep learning algorithm with convolutional neural networks for pose estimation and key point detection. The software integrated multiple architectures providing robustness across radiographic scenarios. Only key points with a confidence score above 50 (out of 100) were used for measurements [23]. Extensive training on 773 lateral and 909 dorsoplantar finely annotated foot X-rays enabled BoneMetrics to attain high accuracy and generalization in the version used.

#### 2.3.2. AI Screening

**Figure 1:**
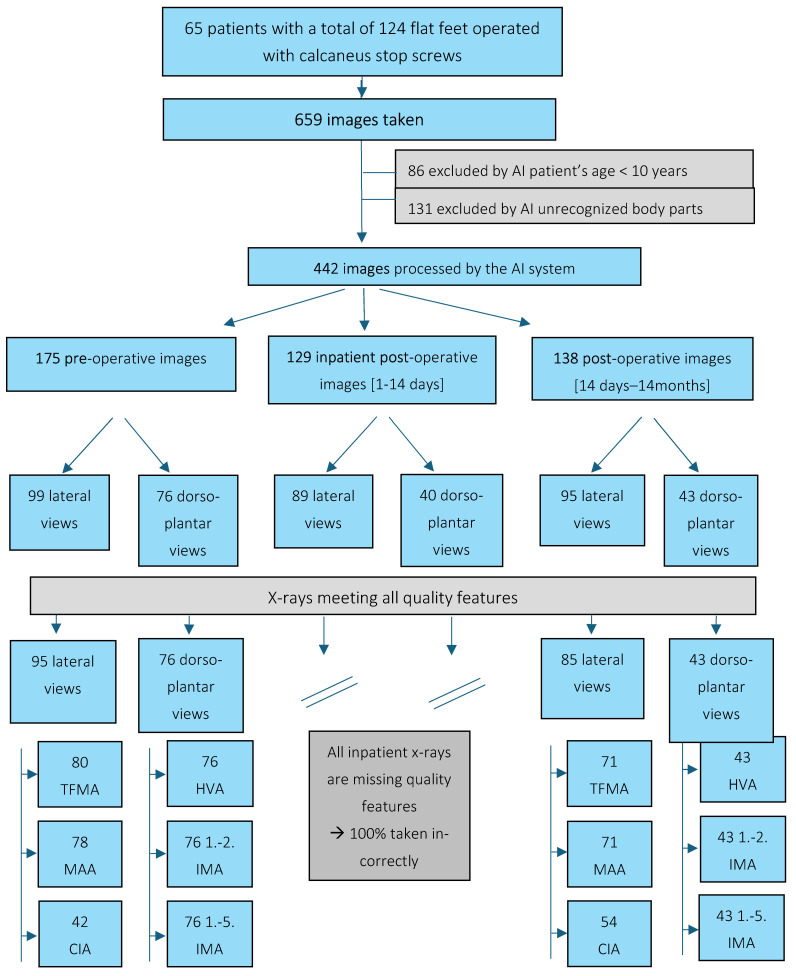
Flow chart showing data collection. The complete set of X-ray images is presented, along with the excluded images by AI and the relevant criteria for this exclusion. Subsequent to this, the images are distributed into different time points and planes before and after the application of the manual quality assessment. This is succeeded by an enumeration of the angles that could be measured by AI. On X-ray in the lateral plane, the AI was programmed to measure the talus-1st metatarsal angle (TMFA), as well as the medial arch angle (MAA) and the calcaneus inclination angle (CIA). The hallux valgus angle (HVA), the 1st–2nd metatarsal angle (1.–2. IMA) and the 1st–5th metatarsal angle (1.–5. IMA) were measurable on X-rays in the dorsoplantar plane.

All image data were fed into the BoneMetrics AI system (Gleamer, Saint Mandé, France) for automated evaluation. Of the 659 images, 422 could be analyzed by BoneMetrics. The software’s categorical exclusion criteria were age under 10 years and misclassification of the body region by the pre-algorithm of BoneMetrics, which led to non-processing in 85 and 131 cases, respectively (Figure 1). In contrast, manual measurements were possible on X-rays of children under 10 years and those with misclassified body parts that could not be analyzed by AI (Figure 1). The analyzed data sets comprised 175 pre-operative images, 129 images from the post-operative interval during the inpatient stay, and 138 images from the post-operative interval during the outpatient stay. After assessing all radiographs for compliance with quality criteria, 95 pre-operative and 85 post-operative outpatient images in the lateral plane, and 76 pre-operative and 43 post-operative outpatient images in the dorsoplantar plane were included in the analysis.

### 2.4. Quality Criteria

Because of the limited load-bearing capacity of the feet, radiographs taken within the first two weeks after surgery (inpatient) were acquired in a non-weight-bearing position. As measurements differ between weight-bearing and non-weight-bearing radiographs, only standing weight-bearing images were suitable for reliable angle assessment, resulting in the exclusion of all inpatient radiographs (Figure 1) [3,4,6,26,27].

Additional inclusion criteria required the preparation of the patient which included a legitimate indication for the X-ray, the patient identification, the labeling of the body part and attaching the gonad protection. For image capture, all relevant anatomical landmarks needed to be fully visible, including the ankle joint and that the foot be positioned in neutral-zero with the heels and toes aligned at the same level [26,27,28]. The foot should be displayed without superimposition. Casts were not permitted [29]. Particularly for lateral radiographs, the X-ray beam had to be projected parallel to the floor, with the longitudinal axis of the foot parallel to the film [27], and the central beam directed at the third metatarsal bone. The lateral border of the foot was positioned on a small 45° wedge pillow. For dorsoplantar radiographs, the central beam was aimed at the midfoot vertically. For children from 8 to 18 years, the image was taken with 50 kV/1.4−1.8 mAs. All this information was clearly defined in the standard operation procedures (SOPs) for X-rays.

### 2.5. Anatomic Definitions

**Figure 2:**
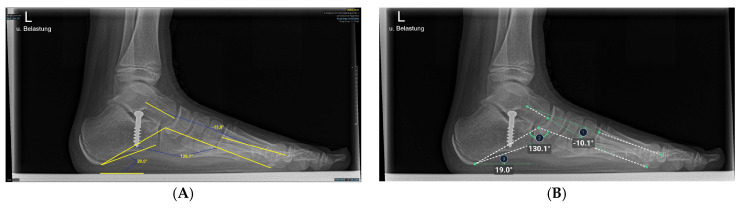
Illustration of talus-1st metatarsal angle, medial arch angle, and calcaneus inclination angle measurements on X-ray images in the lateral plane. (**A**) Manual measurement exemplary. The same X-ray image was processed by the AI. (**B**) Angles calculated by AI.

**Figure 3:**
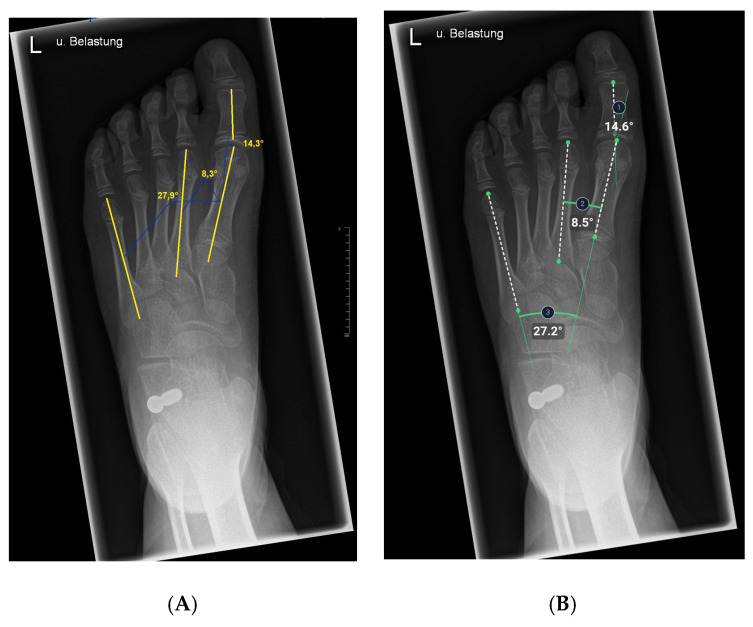
Illustration of hallux valgus angle, 1st–2nd metatarsal angle, and 1st–5th metatarsal angle measurements on x-ray images in the dorsoplantar plane. (**A**) Manual measurement exemplary. The same x-ray image was processed by the AI. (**B**) shows the angles calculated by AI.

At this point, the AI system used was able to measure three angles each of the lateral and dorsoplantar projection (Figure 2 and Figure 3). The talus-1st metatarsal angle and all three angles in the dorsoplantar plane were defined by the intersection of axes. Axes for angle measurements were mechanically defined as lines connecting the center of the bone head and the center of the bone base [30,31]. The talus-1st metatarsal angle was determined by the intersection of the axis of the first metatarsal and a line intersecting the head and neck of the talus [23,32]. (Figure 2). The medial arch angle was measured by a line joining the lowest point of the medial sesamoid with the lowest point of the talonavicular joint and from there a line to the lower edge of the calcaneus [23,32]. The calcaneus inclination angle was defined by the intersection of a line drawn from the inferior cortex of the calcaneus to the inferior corner of the calcaneus, which belongs to the calcaneocuboid joint with the horizontal axis [23]. Physiological values were −4° to 4 ° for the talus-first metatarsal angle. For 8-year-old children, 5 ° was given as the standard values at this age [6]. The normal value was 120 ° to 130° for the medial arch angle and 11 ° to 24 ° for the calcaneal inclination angle on X-rays of immature children [6,33].

The hallux valgus angle is determined by the axis of the first metatarsal bone that of the proximal phalanx [23,32].

Having intersected the axis of the first metatarsal bone with the axis of the second metatarsal bone, the 1st-2nd metatarsal angle was obtained [23,32].

The intersection of the axes of the 1st metatarsal and 5th metatarsal resulted in the third and last angle examined, the 1st-5th metatarsal angle [23].

### 2.6. Statistical Analyses

The data analysis was performed using SPSS Statistics (version 29.0.0, IBM, New York, NY, USA), Excel (version 2408, Microsoft Office LTSC Professional Plus 2024, Microsoft Corporation Redmond, WA, USA) and GraphPad Prism (version 9.5.1, GraphPad Software, San Diego, CA, USA).

Means, standard deviations (SD), and range (minimum and maximum) were presented, as well as odds ratios (OR) and 95% confidence intervals (CI). Statistical significance was set at *p* < 0.05.

In order to compare the AI values to the manual measurements, the normal distribution of all angle pairs was assumed graphically or mathematically using the Shapiro–Wilk test. Their deviation was quantified using the paired *t*-test and visualized via Bland–Altman plots. The influence of various parameters on the measurement accuracy of the AI was analyzed by multiple linear regression model. For this purpose, the difference between the AI-generated measurements and manually measured angles (in degrees) was considered the dependent variable. The independent variables comprised gender and age at the time of X-ray, the presence of an accessory navicular bone (OTE/ONC), and the presence of any additional clinically or radiologically noticeable foot pathology.

A multiple logistic regression analysis was performed to investigate factors influencing the measurability by AI. The dependent variable was defined as measurability. The independent variables corresponded to those of the linear regression analysis, supplemented by the manually determined angle, which depicts the severity of the flat foot deformity.

## 3. Results

### 3.1. Demographics

The patient characteristics reveal 69% male and 31% female patients with an average pre-operative age of 11.5 ± 1.1 years and post-operative age of 12.2 ± 1.5 years (Table 1). A total of 42 patients (72.4%) displayed an accessory navicular bone at least on one foot while 19 patients (32.8%) showed an additional foot pathology.

### 3.2. Comparison Between Manually and AI Generated Angle Measurements

The greatest difference between the measurement methods was found for the talus-1st metatarsal angle. As Table 2 shows, the AI-generated measurement was on average 6.1° (95%-CI: −7.14 bis −5.14; *p* = 0.001) greater than the manual measurement on the pre-operative X-ray image. Even though the AI-generated angles indicated a less severe flat foot than the manually measured angles, the results of both measurement methods confirmed the presence of a flat foot. The mean difference on the post-operative X-ray image amounted to 2.8° (95%-CI: −3.79 −1.81; *p* = 0.001). In both measurement methods, the measurement of this angle showed an elevation of the arch of the foot (Table 2).

For the medial arch angle, the AI generated calculation was on average 1.6° smaller than the manual measurement on the pre-operative X-ray image (95%-CI: 1.03 bis 2.23; *p* = 0.001). Also, post-operatively, the AI generated calculation was 0.5° smaller on average (95%-CI: 0.11 bis 0.93; *p* = 0.013). Again, manual measurements showed a slightly higher severity of the flat foot than AI generated values pre- and post-operatively. An improvement of the arch could be confirmed by both measurement methods (Table 2).

The measurements of the calcaneus inclination angle, in contrast, were not significantly different from each other. Pre-operatively, the AI calculation was in average 0.1° and post-operatively 0.2° smaller than the manual measurement. This angle hardly improved post-operatively in both measurement methods (Table 2).

The difference in the measurement methods in the dorsoplantar view was small but statistically significant for all angles measured on pre-operative X-ray images. The hallux-valgus angle was 0.4° greater when measured by AI (*p* = 0.002, 95%-CI [0.15; 0.66]), the 1st–2nd metatarsal angle was 0.3° smaller when AI generated (*p* = 0.011, 95%-CI [0.06; 0.47]) and the 1st–5th metatarsal angle showed a difference of 0.3°, and was slightly higher in the manual measurement (*p* = 0.009, 95%-CI [0.08; 0.52]).

On the post-operative X-ray images, the measurement differences in the hallux-valgus angle and the 1st–2nd metatarsal angle were not statistically significant and too small to be clinically relevant. The 1st–5th metatarsal angle was on average 0.4° (*p* = 0.006, 95%-CI [0.13; 0.74]) smaller in AI generated measurement.

**Figure 4:**
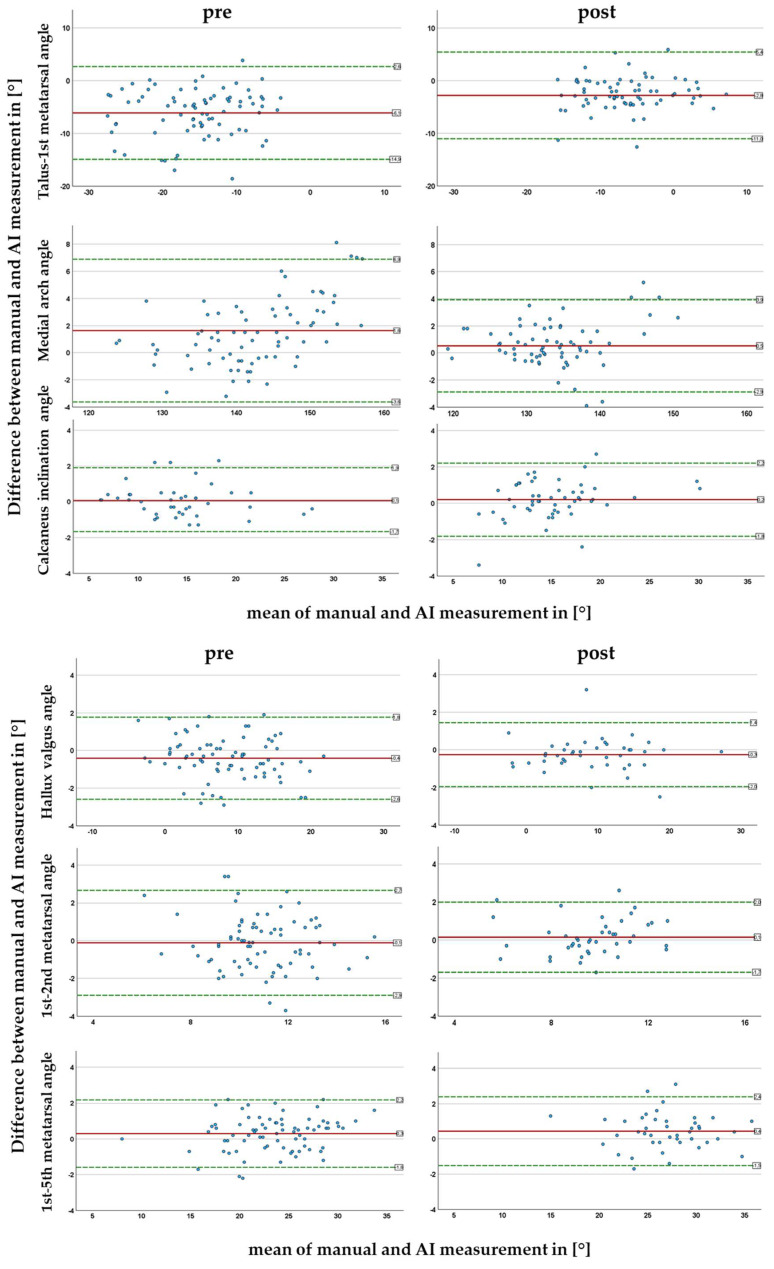
Comparison between manually and AI generated angle measurements in conventional radiological examinations infantile flat feet visualized in Bland–Altman plots. The Bland–Altman plots show the deviations between the measurement methods of each X-ray image in blue. The mean difference between the two measurement methods is shown in red. The green lines show the upper and lower fluctuation range. They are calculated by the mean value ± 1.96 times the standard deviation of the differences. Upper part: lateral plane, lower part AP plane.

In order to illustrate the deviations between the manually and AI-generated measurement, to compare pre and post clearly and to detect possible trends, Bland–Altman plots were created (Figure 4).

#### 3.2.1. Talus-1st Metatarsal Angle

The mean AI generated measurement showed a systematically larger result than the mean manual measurement of the talus-1st metatarsal angle. This led to a negative bias of the two measurement methods in the Bland–Altman plot in pre- as well as post-operative measurements (Figure 4). When investigating the reason for the large bias, we noticed that the AI measured the axis of the first metatarsal angle was measured steeper than our observer.

The mean difference was post-operatively 3.3° smaller than pre-operatively. In one case, a difference between the measurement methods of −28.8° was found in the post-operative data set. In addition to this one conspicuously large difference in the methods, no other measurement pair indicated a negative effect on the measurement of this angle the X-ray image with a screw implanted. The subsequent image interpretation revealed no relevant correlation between the mean angle depicted on the *x*-axis and the measurement discrepancy for pre- and post-operative analysis.

#### 3.2.2. Medial Arch Angle

For the medial arch angle on the other hand, a slight trend was found. The difference between methods tended to get larger as the average mean medial arch angle increased. In this angle, the severity of the flat foot seemed to influence the accuracy of the AI-generated measurement negatively.

Additionally, it was found that the systematic bias decreased by 1.1° (67.8%) when comparing the post-operative Bland–Altman plot to the preoperative one.

#### 3.2.3. Calcaneus Inclination Angle

A mean difference between the measurement methods, too small to be clinically relevant, was detected for the calcaneus inclination angle in pre- as well as post-operative measurements.

Postoperatively, the AI seemed to calculate lightly smaller values when the flat foot was more severe and slightly higher values when the mean calcaneus angle was more physiological (Figure 4).

#### 3.2.4. Hallux Valgus, 1st–2nd and 1st-5th Metatarsal Angles

In the dorsoplantar view, a systematic deviation of less than half of a degree of the measurement methods of the pre- and post-operative angles on X-ray images were not clinically relevant (and most likely be considered as random). The values scattered around the bias line; no trend was visible. The bias as well as the deviation were comparable between measurements on pre- and post-operative X-rays. An influence of the stop screw on the quality of angle measurements in dorsoplantar X-rays was not proven.

### 3.3. Factors Influencing the Quality of AI Measurements

There were non-significant effects of gender, age, accessory navicular bone/os tibiale externum and additional foot pathology on the difference in talus-1st metatarsal angle between the two measurement methods, both pre- and postoperatively. The presence of an Os naviculare cornutum/Os tibiale externum on the postoperative X-ray tended to reduce the difference by 2.7° (*p* = 0.051; 95% CI: −5.40 to 0.16). Contrary to expectations, the presence of this anatomical variation did not have a negative influence on the AI-generated measurement.

Gender significantly influenced the measurement difference in the preoperative medial arch angle. In boys, it was 1.9° higher than in girls (*p* = 0.006; 95% CI: 0.58 to 3.28). In addition, the difference between the measurement methods decreased significantly by 0.6° per year with increasing age (*p* = 0.026; 95% CI: −1.19 to −0.08). The remaining variables in the linear regression analysis had no significant influence on the measurement difference in the preoperative medial arch angle. Gender and age influenced the difference between the measurement methods of the postoperative medial arch angle significantly. The measurement difference was 1.1° greater in boys than in girls (*p* = 0.020; 95% CI: 0.17 to 1.93) and decreased by 0.3° per year with increasing age (*p* = 0.021; 95% CI: −0.61 to −0.05).

None of the influencing factors examined had a significant effect on the difference between the measurement methods for the calcaneus inclination angle, neither pre-, nor post-operatively.

The influence of gender, age, and the presence of an ONC/OTE or additional foot pathology on the difference between manual and AI measurement was low and statistically insignificant for all angles examined in the dorsoplantar plane, both pre- and post-operatively. Only in the pre-operative 1st-2nd metatarsal angle was the measurement difference significantly greater by 0.5° when an ONC/OTE (*p* = 0.028, 95% CI [0.06; 1.03]), as well as in the presence of additional foot pathology (*p* = 0.010; 95% CI [0.127; 0.955]).

### 3.4. Factors Influencing the Measurability by AI

**Figure 5:**
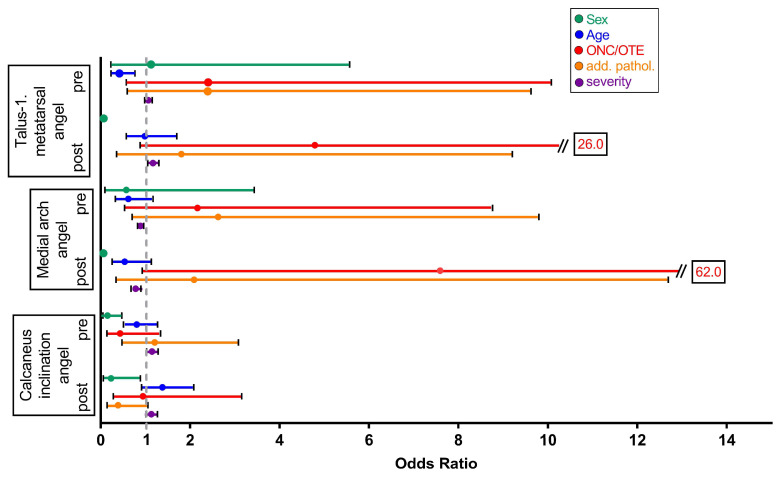
Factors influencing the measurability by AI. Association of measurability of angles in lateral plane with age, sex, the presence of ONC/OTE or an additional foot pathology and the manually measured angle calculated by multiple logistic regression analysis. Presented are the calculated odds ratios (OR) as dots for the chance of measurability in dependence on the factors mentioned above and their 95% CI as lines. OR ≠ 1 indicates a correlation. A 95% CI Ø 1 indicates statistical significance. ONC os naviculare cornutum, OTE os tibiale externum. Accessory navicular bone.

Possible reasons for the lack of measurability of angle on correctly taken X-rays were examined using a multiple logical regression model (Figure 5). Measurability by AI was reduced when key-points could not be determined with a confidence over 50%. Only missing AI calculations on lateral plane images were considered as all images in dorsoplantar plane could be fully examined by AI.

The talus-1st metatarsal angle was not measured on 15 of 95 pre-operative X-ray images, especially often in older children. If age increased by 1 year, the chance of this angle being measurable by AI decreased by 58% (95% CI = 0.230 to 0.767; *p* = 0.005). The influence of the other parameters (gender, presence of an accessory navicular bone (ONC/OTE), additional pathology, and severity) on the chance of measurability of the pre-operative talus-first metatarsal angle was not significant.

Postoperatively, the measurement of this angle was missing on 14 of 85 X-rays. More precisely, in 14 of 85 X-rays, the AI was not able to determine the necessary points confident enough. The chance of measuring the post-operative talus-1st metatarsal angle did not result in any statistically significant values for gender, age, the presence of ONC/OTE, or additional foot pathology. In contrast to this, the severity of the flat foot had a statistically significant negative influence on measurability. If the manually measured angle increased by 1°, the chance of the angle being measurable by AI increased by 17% (95% CI: 1.051 to 1.302; *p* = 0.004).

The AI was able to determine the medial arch angle in 79 of the 95 pre-operative X-ray images. While female gender had a significant positive influence on the measurement quality of this angle, neither gender nor age, nor the presence of chronic overload signs or additional foot pathology had a significant influence on measurability. However, if the manually measured angle increased by 1°, the chance of the angle being measurable by AI decreased by 10.8% (*p* = 0.003; 95% CI: 0.824 to 0.964).

While the medial arch angle could be determined manually in all 85 postoperative images meeting the quality criteria, the AI only measured it in 71 X-ray images. Gender, age, the presence of ONC/OTE, or additional foot pathology could not significantly explain the absence of the remaining 14 AI-generated measurements. Only an increase of 1° in the manually measured angle reduced the chance of the angle being measurable by AI by 21.8% (95% CI: 0.680 to 0.899; *p* = 0.001).

In 53 of the 95 correctly recorded preoperative X-ray images sent to the AI for processing, the calcaneus inclination angle could not be determined. Logistic regression analysis revealed that boys were 85.8% less likely than girls to have the angle determined by AI, OR = 0.152, (95% CI: 0.049 to 0.472; *p* = 0.001). The severity of the flat foot also contributed to the images not being measured: if the manually measured angle increased by 1°, the chance of the angle being measurable by AI increased by 14.9% (95% CI: 1.027 to 1.284; *p* = 0.015). The other independent variables examined had no significant influence on the measurability of the calcaneus inclination angle.

Postoperatively, the AI did not measure the calcaneus inclination angle on 31 of 85 X-rays. Gender had a significant influence on the measurability here. Male patients had a 76.9% lower chance of the angle being determined by AI (95% CI: 0.060 to 0.997; *p* = 0.033). In addition, a correlation was found between a 1° increase in the manually measured angle and a 13.2% increase in the chance of the angle being measurable by AI (95% CI: 1.011 to 1.268; *p* = 0.032).

In the dorsoplantar view, the hallux valgus angle, the 1st–2nd metatarsal angle, and the 1st–5th metatarsal angle could be measured on all preoperative (n = 76) and postoperative (n = 43) X-rays. Therefore, a logistic regression analysis was not necessary.

### 3.5. Influence of Stop Screws on the Measurability of the Angles

**Figure 6:**
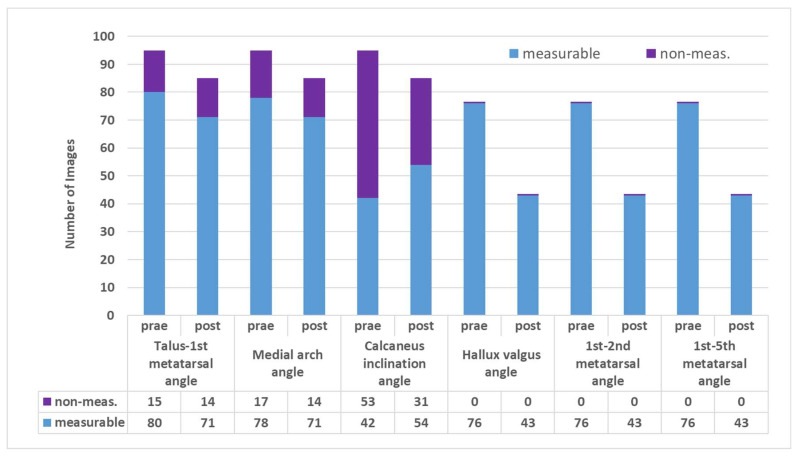
Number of measurable angles on pre- and post-operative X-ray images.

In the lateral view, the AI was unable to calculate all manually measurable angles (Figure 6). The proportion of the measurability of the talus-first metatarsal angle and the medial arch angle between the pre- and postoperative X-ray images were only slightly different from each other with less than 1% difference for the talus-first metatarsal and 1.4% for the medial arch angle. The rate of X-ray images with the calcaneus inclination angle being measurable was even 19.3% higher for post- than for pre-operative X-rays.

In the dorsoplantar view, all three angles were measurable on all X-ray images for both the pre- and post-operative views. This indicates that the measurability was 100% throughout. Consequently, the hypothesis that the calcaneus stop screw had a detrimental effect on the measurability of the angles examined by AI could be rejected.

### 3.6. Proportion of Measurable X-Rays Depending on Quality Features

The proportion of X-rays in the lateral view missing quality features was even higher than the measurable X-rays meeting all quality features (Table 3). The difference in the number of AI-generated measurements between X-rays meeting and missing quality features amounted to 1.5% for the talus-1st metatarsal angle and 4.6% for the medial arch angle. When it came to measuring the calcaneus inclination angle, the difference was especially noticeable, at 16.6%. Furthermore, this angle could only be measured in 56.5% of the imported images, which was lower than for the other two angles in this plane (84.5%). The proportion of X-rays in the dorsoplantar view missing quality features was equal with the measurable X-rays meeting all quality features.

All in all, the AI did not recognize incorrectly recorded X-ray images. Consequently, the system did not deny measuring those angles.

## 4. Discussion

This study showed that the AI software BoneMetrics had been well trained for the measurement of all three measurable angles on X-rays in the dorsoplantar view.

In the lateral view on the other hand, the AI calculated significantly different values for the talus-1st metatarsal angle and the medial arch angle. Contrary to our expectations, the measurement difference in the talus-1st metatarsal angle was higher in older children. As the postoperative measurement was not negatively influenced by age, we interpreted this finding as a statistical error.

The calcaneus inclination angle could only be measured by AI on a low number of X-rays. Especially on X-rays of younger children and more severe flat feet, the chance of measurability of the calcaneus inclination angle decreased.

Improvement of measurement quality in the lateral plane and key point identification was therefore considered as necessary before relying on AI in medical diagnostics.

The AI tool was not found to be less useful in measuring angles on post-operative than pre-operative X-rays. Therefore, implanted calcaneus stop screw could not be defined as a negative influencing factor on neither the measurability nor the quality of AI generated measurement.

When assessing the validity of AI-derived values, it is important to remember that even human raters show variability. Bock et al. [19] demonstrated only high—but not excellent—correlation between observers for lateral foot angles. Seung Min Ryu et. al. [25] and Lassalle et al. [23] even reported stronger agreement between human–AI than human–human pairs. To fully appraise AI accuracy, differences between AI and manual measurements should be benchmarked against interobserver variability.

The proportion of X-rays meeting all quality features being measurable by AI was not bigger than the proportion of X-rays missing quality features. BoneMetrics was not able to distinguish reliably between correctly and incorrectly taken X-rays. The capacity to identify missing quality criteria on X-rays, as evidenced by the expertise of a medically trained individual, cannot be replaced by artificial intelligence.

In the lateral view, AI-based measurements showed the greatest limitations. Values for the talus–1st metatarsal and medial arch angle deviated significantly from manual measurements. AI consistently produced steeper axes of the first metatarsal leading to systematically more physiological values for the talus-1st metatarsal angle. This phenomenon persisted despite identical axis definitions being applied in both methods, namely the mechanical axis, which is defined as a bisecting line between the head and the base of the bone [30]. Unfortunately, some other AI systems used the anatomical definition of the axis of the first metatarsal [24] or did not mention which kind of definition they used [21]. As inconsistent measurement methods due to imprecise definitions of the measured parameters lead to inaccurate data, we did not compare our findings to other AI systems [34]. Since the talus–1st metatarsal angle correlates with symptoms [35] and is used to assess flexibility of flatfoot [36], algorithmic refinement across AI systems and additional training on pediatric flatfoot cases appear clinically relevant. Our post-operative measurements as well as another study caried out with BoneMetrics showed a smaller bias for this angle than our pre-operative measurements [23]. Possible reasons could be the older mean age and more physiological angle measurements in Louis Lassalles study and in our postoperative data set—even though our linear regression analysis did not reveal higher age as a significantly positive influencing factor on measurement accuracy of the AI-generated talus-1st metatarsal angle [23]. As the clinical validation study was conducted on mostly adult feet with a mean age of 51 years for lateral radiographs, improvement is expected for the next version when a higher amount of children’s feet can be included [23]. Patton et al. also demonstrated high agreement between convolutional neural network (CNN)-based AI and manual measurements in young children, also suggesting that improved training data could also enhance BoneMetrics’ performance in pediatrics [21].

For the calcaneus inclination angle, AI achieved successful measurements in only a low number of cases—particularly rarely in pre-operative X-rays, in younger children and in severely deformed flat foot. Overlapping bony structures, such as the navicular bone or a low-lying talus, often prevented AI from identifying the inferior corner of the calcaneus that belonged to the calcaneocuboidal joint. Human raters, by contrast, had little difficulty delineating these structures. Lassalle et al. reported failed AI measurements in only 3% of radiographs [23]. This discrepancy may be explained by the higher prevalence of pathological feet in our sample.

The measurability of three angles in the lateral view was negatively influenced by the severity of the flat foot. This finding could be explained by the composition of the AI’s training data set. It consisted of approximately 80 cases with flat feet. In the clinical validation study by Louis Lassalle, only 17 patients presented with flat feet [23].

The gender-dependency of the medial arch angle and the calcaneus inclination angle could be explained by hormonal influences on the bone maturation. Firstly, the time when puberty set on was found to be two years earlier in girls than in boys. Secondly, studies showed that estrogens were relevant for bone age progression and growth plate fusion. Even before puberty, girls were found to have higher estrogen levels than boys, leading to earlier bone maturation [37].

In the dorsoplantar projection, BoneMetrics performed considerably better. All three angles could be measured consistently, with smaller deviations compared to earlier software versions, indicating ongoing algorithmic improvement [23]. To evaluate its applicability in clinical practice, however, measurements must also be validated in pathological feet. The talus-1st metatarsal angle can be determined in both the lateral and dorsoplantar planes [6], and their sum form the so-called TMT index, common in assessment of flatfoot deformity [14]. In the lateral plane, the navicular to floor distance (NFD), the cuboid to floor distance (CFD) as well as the calcaneus-metatarsale 1 angle by Costa Batani provide further evaluation of the longitudinal arch. It was shown that they correlate with clinical assessments [14,26,30,38].

A further limitation identified was the AI’s inability to distinguish between radiographs that met quality standards and those that did not. This is problematic, as inadequate positioning or non-weight-bearing imaging may lead to misinterpretation [18,26,27]. Weight-bearing reflects actual foot function, revealing malposition, functional information, and bone relationships that are not visible otherwise [18,26]. Consequently, reliable angles for diagnosing chronic foot deformities can only be obtained from weight-bearing radiographs [3,26,27]. Other AI powered tools could already assess image quality and identify issues effectively [39]. As soon as an automated image quality assessment tool is implemented in BoneMetrics, the tool is expected to provide more reliable measurements in real time leading to a greater reliable number of comparable angles [39]. A future study should therefore be larger in sample size and in prospective style enabling the examination of even more possibly influencing variables. It should also include further data sets to validate inter- and intra-observer reliability.

As pediatric patients are particularly vulnerable to radiation exposure, unnecessary radiographs must be avoided [28]. According to the current literature, weight-bearing on the foot was possible after a mean period of 8 to 14 days after surgery [14,15]. Therefore, the inpatient radiographs excluded in our study were considered non-weight-bearing and therefore unsuitable for flat foot quantification [28,40]. Given that intraoperative radiographs already confirm screw placement, further routine inpatient imaging may be redundant and should be critically reconsidered [14].

A potential limitation of this study is the uncertainty regarding the weightbearing status of the radiographs. Although they were intended to be acquired under weight-bearing conditions, it cannot be definitively confirmed that this was the case for all images. This uncertainty may affect the accuracy and consistency of the radiographic measurements. Staff and device changes over the years may also have impacted the consistency of the X-ray imaging technology. However, we interpreted the influence as negligible as the SOPs were strictly controlled. Furthermore, as previously mentioned, manual measurements were performed by a single rater with adequate medical training, under the supervision of both a senior pediatric orthopedic surgeon and a senior radiologist. Nonetheless, inter-rater variability remains a relevant concern in manual measurements. As shown by Bock et al. [19], correlations between observers for lateral foot angles were high but not excellent, indicating a degree of subjectivity that could influence the reliability of the results.

Currently, BoneMetrics excludes radiographs from children under the age of ten. The consequence of this was the exclusion of a considerable number of cases in the present study. Calcaneus stop screw arthroereisis is recommended from the age of nine for symptomatic flat foot [17]. Further training of the algorithm to extend applicability to nine-year-olds would substantially increase the amount of comparable data for a future study.

The application of exclusion criteria by AI (age under 10, unrecognized body parts) and our exclusion of incorrectly taken radiographs resulted in a substantial reduction in the sample size. Unfortunately, incompletely documented information on possible influencing factors such as BMI, pain, range of motion, conservative treatment, previous operations, and leg axis pathologies on AI measurement led to exclusion of these criteria in our regression analysis. We also excluded genetic disorders that could cause ligamentous laxity from our regression analysis since it could not be assumed that the diagnosis had already been confirmed in all affected children in early age.

## 5. Conclusions

Even though the indication for the surgical implantation of calcaneus stop screws is orientated on clinical symptoms, angle measurements help quantify the severity and the therapeutic outcome. The use of the current version of Gleamer’s AI requires manual remeasurement of the angles. A growing impact of automated angle measurements is expected with continuous training of the system. Automated evaluation of surgical results and the evaluation of surgical outcomes for each surgeon individually would require significant training, as well as access to clinical data.

A future study should therefore be larger in sample size and in prospective style enabling the examination of even more possibly influencing variables. It should also include further data sets to validate inter- and intra-observer reliability.

The potential value of artificial intelligence in supporting pediatric foot diagnostics may even increase further due to the highly prevalent childhood overweight and obesity number in Germany [41]. With obesity being an important risk factor for pediatric flat feet, the number of pediatric patients presenting with this condition may further increase [6,7,8].

## Figures and Tables

**Table 1:** Patient characteristics. The patients under 10 years were excluded. Patients with partially missing AI generated measurements or X-ray images missing quality features were included in the table. SD standard deviation.

Number of Patients Older than 9 Years	58
Number of operated feet	
Both sides	53
Only right	3
Only left	2
Gender	
Male	40 (69%)
Female	18 (31%)
Age in years	
Pre-OP: mean ± SD	11.5 ± 1.1
Range pre-OP	10–14
Post-OP: mean ± SD	12.2 ± 1.5
Range post-OP	10–15
Foot Pathologies	
Accessory navicular bone (os tibiale externum or os naviculare cornutum)	42
on at least one foot	
Additional foot pathology	
on at least one foot	19

**Table 2:** Comparison of manually and AI-generated measurements of angles. The table shows the mean value in ° of the two methods of measurements, the absolute difference in degrees, and their two-sided *p*-value.

X-Ray Planes	Angle	Time	X-Rays Meeting All Quality Standards and Measurable by AI [n]	Manual Measurement Mean ± SD [°]	AI Measurement Mean ± SD [°]	Difference (Manual—AI) Mean ± SD [°]	*p*-Value
Lateral view	Talus-1st metatarsal angle	pre	80	−18.5 ± 6.8	−12.4 ± 6.4	−6.1 ± 4.5	0.001
		post	71	−7.8 ± 6.0	−5.0 ± 5.8	−2.8 ± 4.2	0.001
	Medial arch angle	pre	78	143.7 ± 9.1	142.1 ± 7.7	1.6 ± 2.7	0.001
		post	71	133.3 ± 7.4	132.8 ± 7.0	0.5 ± 1.7	0.013
	Calcaneus inclination angle	pre	42	14.3 ± 4.8	14.2 ± 5.0	0.1 ± 0.9	0.629
		post	54	15.3 ± 5.6	15.1 ± 4.3	0.2 ± 1.0	0.169
Dorso-plantar view	Hallux valgus angle	pre	76	8.2 ± 5.7	8.7 ± 5.9	−0.4 ± 1.1	0.002
		post	43	9.1 ± 6.3	9.3 ± 6.4	−0.3 ± 0.9	0.062
	1st–2nd metatarsal angle	pre	76	11.1 ± 1.9	10.8 ± 1.7	0.3 ± 0.9	0.011
		post	43	9.8 ± 1.9	9.6 ± 1.8	0.1 ± 0.9	0.312
	1st–5th metatarsal angle	pre	76	23.6 ± 4.6	23.3 ± 4.4	0.3 ± 1.0	0.009
		post	43	27.2 ± 4.2	26.8 ± 4.2	0.4 ± 1.0	0.006

**Table 3:** Proportion of measurable X-rays depending on quality features present. The number of imported X-rays, composed of pre-operative as well as inpatient after surgery and outpatient post-operative images and the percentage of all measurable X-rays are illustrated. A division into the ones meeting all quality features and the ones missing quality features and their proportions was made.

X-Ray Planes	Angle	Imported X-Rays [n]	Measurable X-Rays [%]	X-Rays Meeting All Quality Features [n]	Measurable X-Rays Meeting All Quality Features [%]	X-rays Missing Quality Features [n]	Measurable X-Rays Missing Quality Features [%]
Lateral view	Talus-1st metatarsal angle	283	84.5	180	83.9	103	85.4
	Medial arch angle	283	84.5	180	82.8	103	87.4
	Calcaneus inclination angle	283	56.5	180	53.3	103	70.9
Dorsoplantar view	Hallux valgus angle	161	100	119	100	42	100
	1st–2nd metatarsal angle	161	100	119	100	42	100
	1st–5th metatarsal angle	161	100	119	100	42	100

## Data Availability

The datasets analyzed during the current study are available from the corresponding author on reasonable request due to due to licensing restrictions.

## Abbreviations

The following abbreviations are used in this manuscript:

AI	Artificial Intelligence
ANB	Accessory navicular bone
CI	95% confidence intervals
CFD	cuboid to floor distance
CNN	convolutional neural network
HVA	hallux valgus angle
1.–2. IMA	1st–2nd metatarsal angle
1.–5. IMA	1st–5th metatarsal angle
MAA	medial arch angle
NFD	navicular to floor distance
ONC	Os naviculare cornutum
OP	Operatively
OR	odds ratios
OTE	Os tibiale externum
SD	standard deviations
SOPs	standard operation procedures
TMFA	talus-1st metatarsal angle
TMT	Talometatarsal index
